# Supplementary figures and images for: Distinct S-adenosylmethionine synthases link phosphatidylcholine to mitochondrial function and stress survival
Source: PLoS Biol. 2025 Dec 1;23(12):e3003075. doi: 10.1371/journal.pbio.3003075 (PMC12680360; doi:10.1371/journal.pbio.3003075)

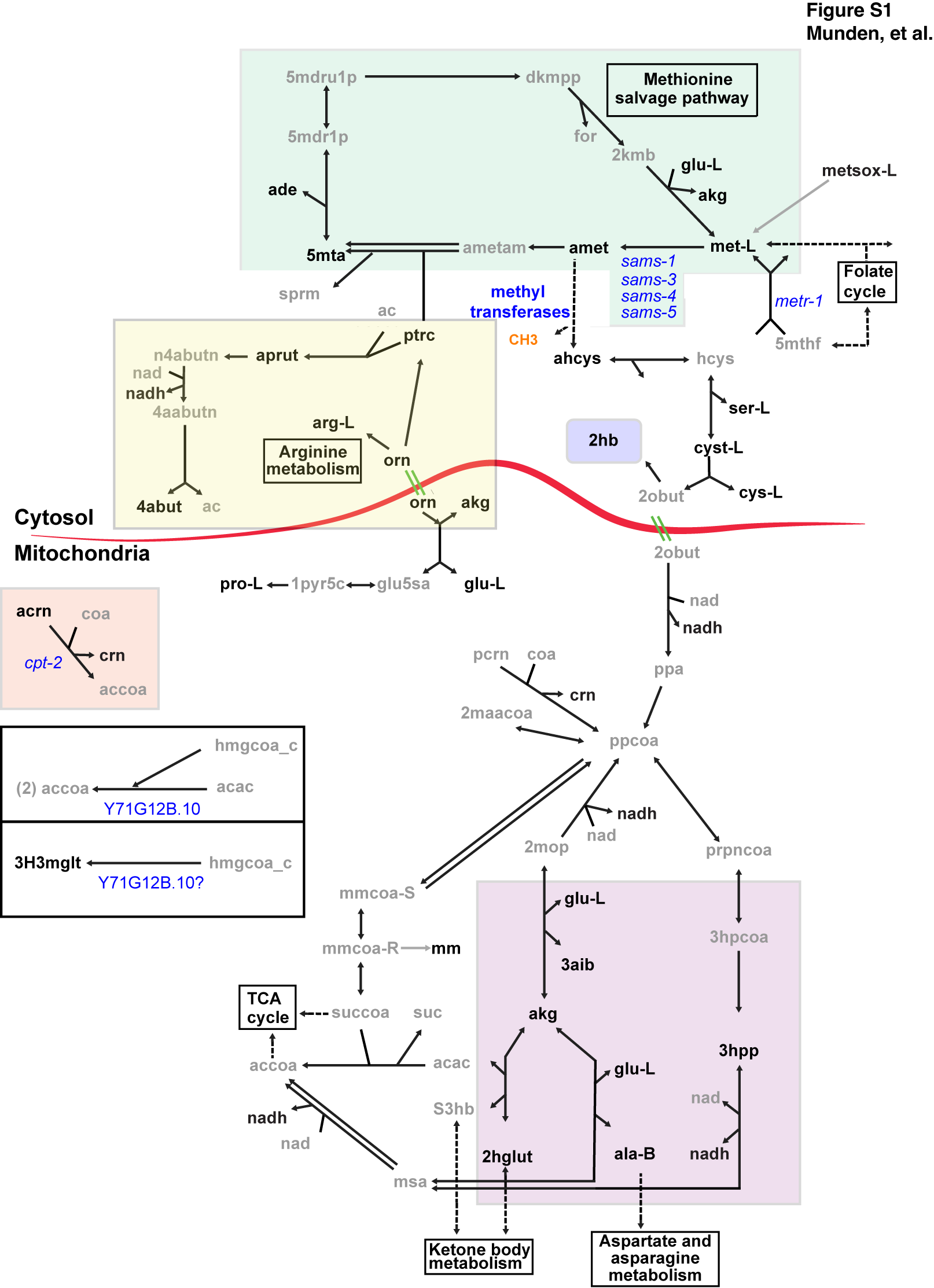

Supplement: S1 Fig — Pathways were adapted from WormPaths [17]. Colored boxes correspond to metabolites shown in individual graphs. Bolded metabolites are represented in targeted metabolomics. (TIF) [file pbio.3003075.s001.tif]

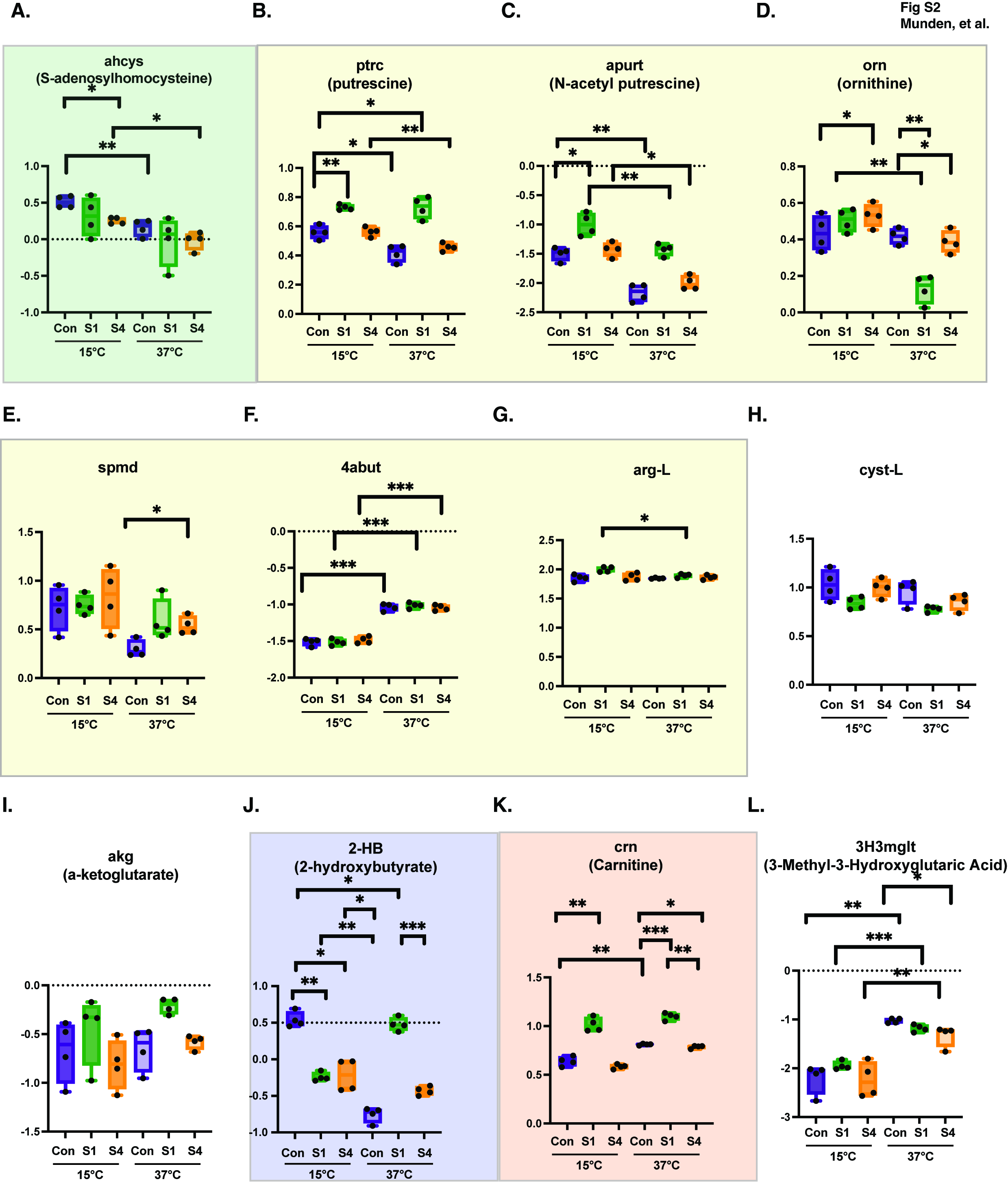

Supplement: S2 Fig — Box and whisker plots showing individual metabolites from targeted metabolomics comparing heat-shocked sams-1 and sams-4(RNAi) animals (A–L). Colored boxes show location of selected metabolites on S2 Fig. Significance was determined by two-way repeated measures ANOVA. ns: q-value ≥ 0.05 *: q-value < 0.05, **: q-value < 0.01, ***: q-value < .001, ****: q-value < 0.0001. Color blocks map to areas on metabolic map (Figs S2, 2 and 3). Underlying data is in S1 Table. (TIF) [file pbio.3003075.s002.tif]

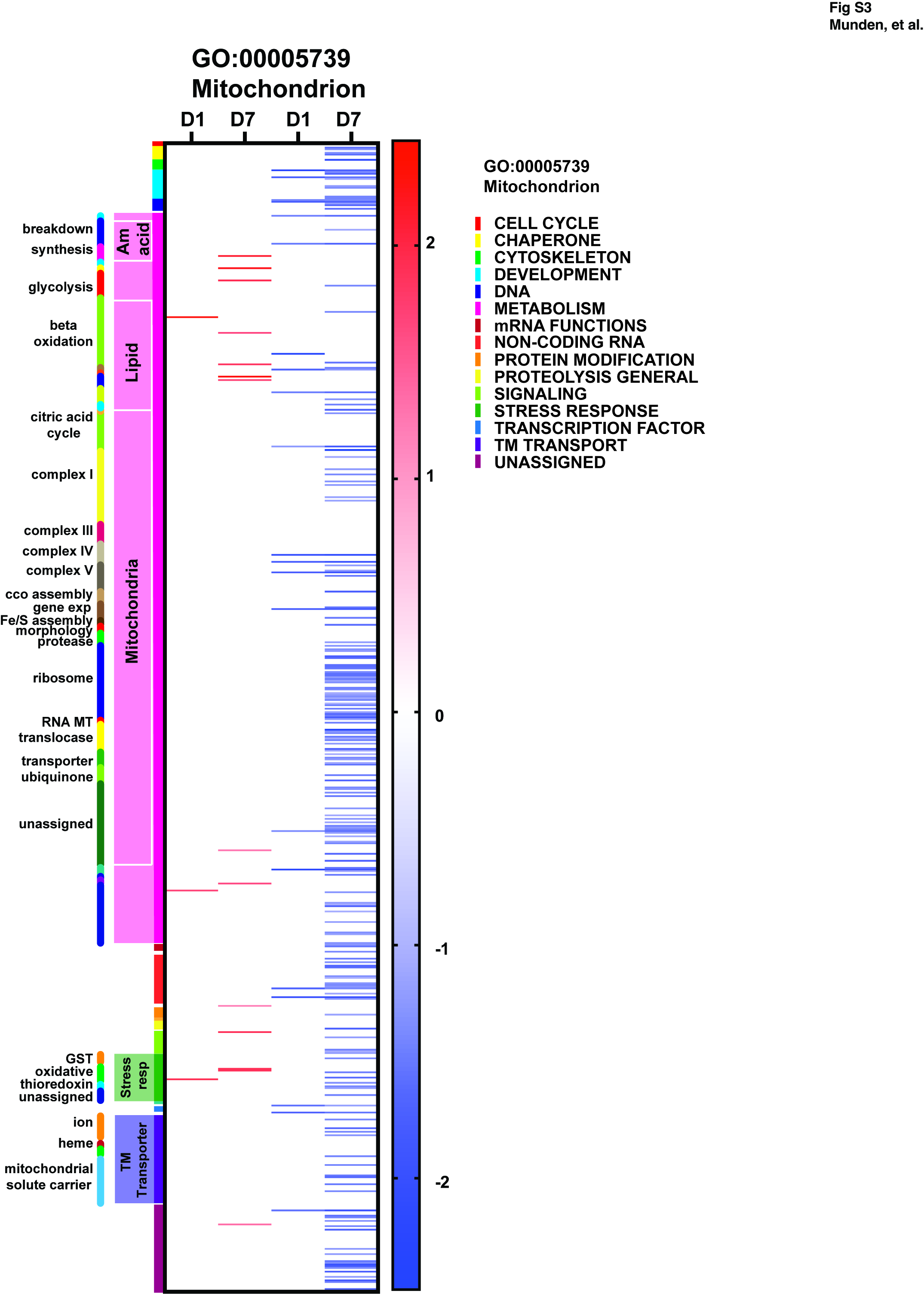

Supplement: S3 Fig — Heat map of GO: 00005739 (Mitochondrion) category. Underlying data is in S2 Table. (TIF) [file pbio.3003075.s003.tif]

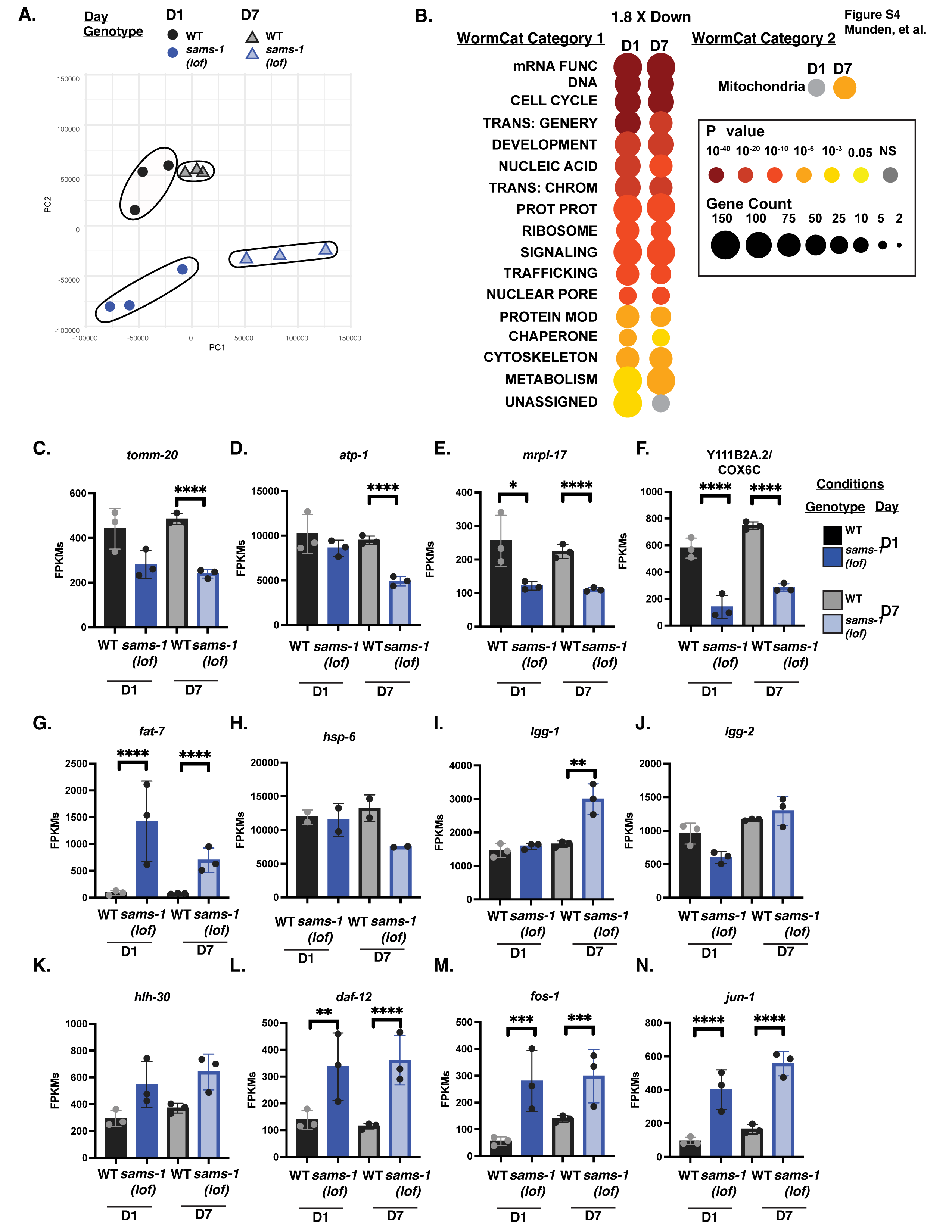

Supplement: S4 Fig — (A) PCA plot showing groupings of three replicates from WT and sams-1(lof) animals at D1 and D7 of adulthood. (B) Bubble chart showing WormCat category 1 and 2 enrichments compared in WT and sams-1(lof) animals at D1 and D7 of adulthood. FPKMs from mitochondrial genes (C–F), fat-7 (G), a lipid metabolic gene that is induced when sams-1 is reduced [66], mitoUPR and autophagy genes (H–K) and other stress-related transcription factors (L–N). Error bars show standard deviation and the p-adjust value calculated by Deseq2 shows significance including a false discovery rate with * p < 0.01, ** p < 0.005, *** p < 0.001. Underlying data is in S3 Table. (TIF) [file pbio.3003075.s004.tif]

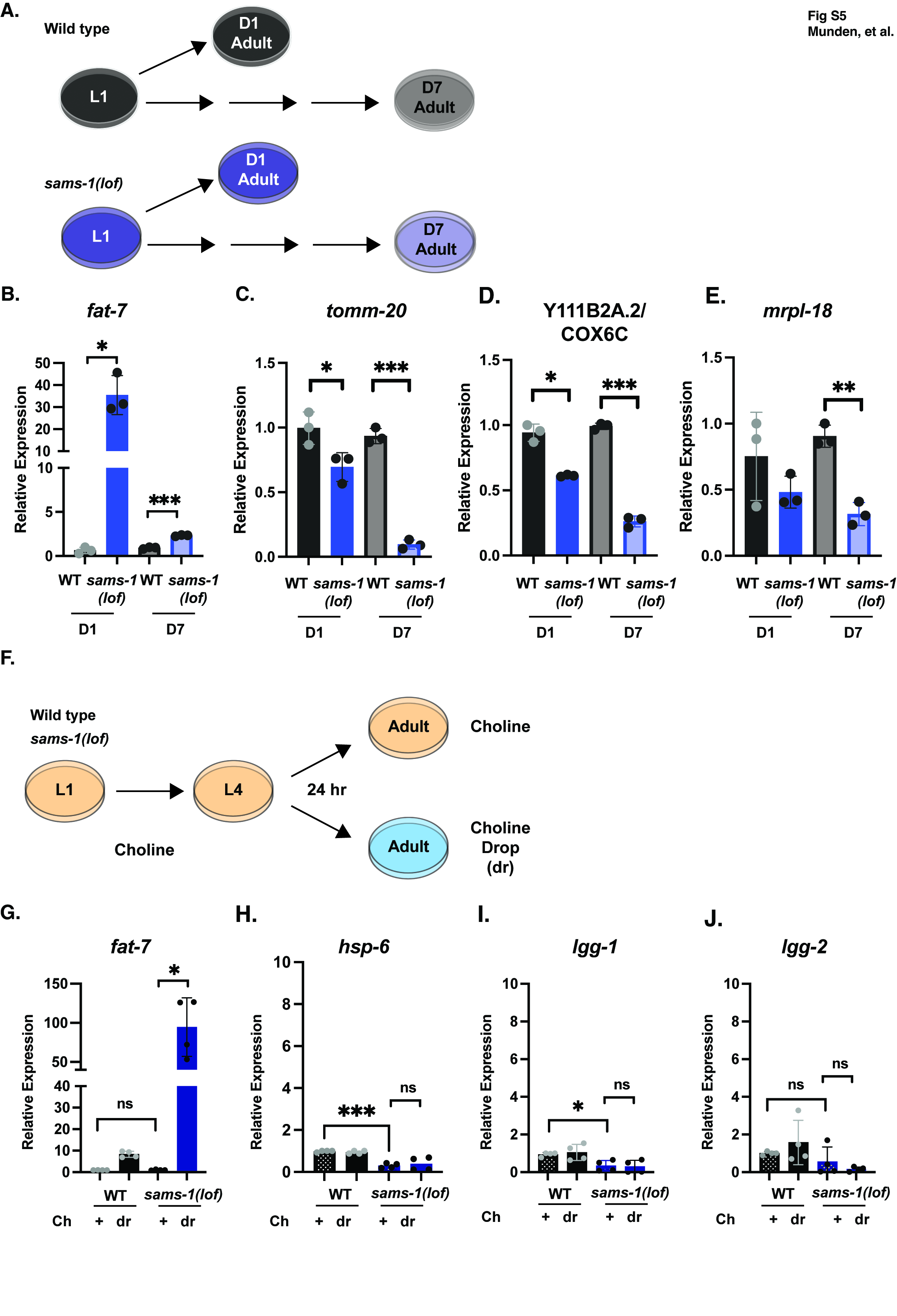

Supplement: S5 Fig — (A) Schematic showing experimental setup for generating populations of D1 and D7 animals for RNA preparation. qRT-PCR comparing fat-7 (B), tomm-20 (C), Y111B2A.2/COX6C (D), and mrpl-18 (E). (F) Schematic showing experimental paradigm for choline drop experiment. qRT-PCR assays expression in animals maintained on choline, or where choline was removed as animals transitioned to adulthood comparing fat-7 (G), hsp-6 (H), lgg-1 (I), and lgg-2 (J). Significance calculated between pairs by the Student’s test with Welch’s correction and shown with * p < 0.01, ** p < 0.005, *** p < 0.001. Underlying data is in S1 Data. (TIF) [file pbio.3003075.s005.tif]

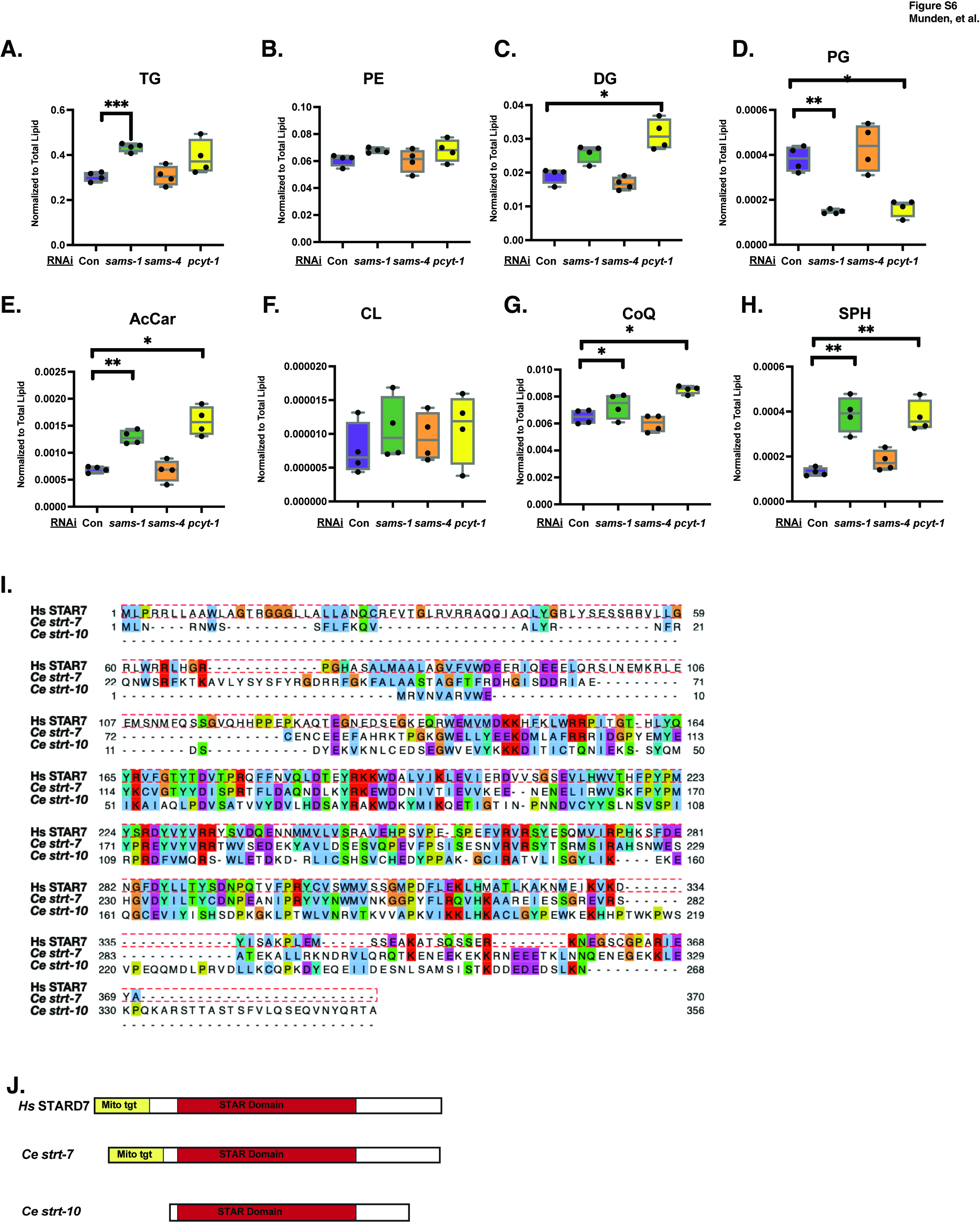

Supplement: S6 Fig — LCMS analysis comparing lipid class levels after sams-1, sams-4 or pcyt-1(RNAi) for TG (triglycerides) (A), PE (phosphatidylethanolamines) (B), diglycerides (DG, C), phosphatidylglycerols (PG, D), Acylcarnatines (AcCa, E), cardiolipins (CL, F), Ubiquinone isoforms (CoQ, G), and sphingolipids (SPH, H). Underlying data is in S4 Table. (I) JalView visualization of a Clustal alignment of C. elegans strt-related proteins with human STAR7. (J) Diagram showing localization of mitochondrial targeting sequences and the STAR domain. (TIF) [file pbio.3003075.s006.tif]

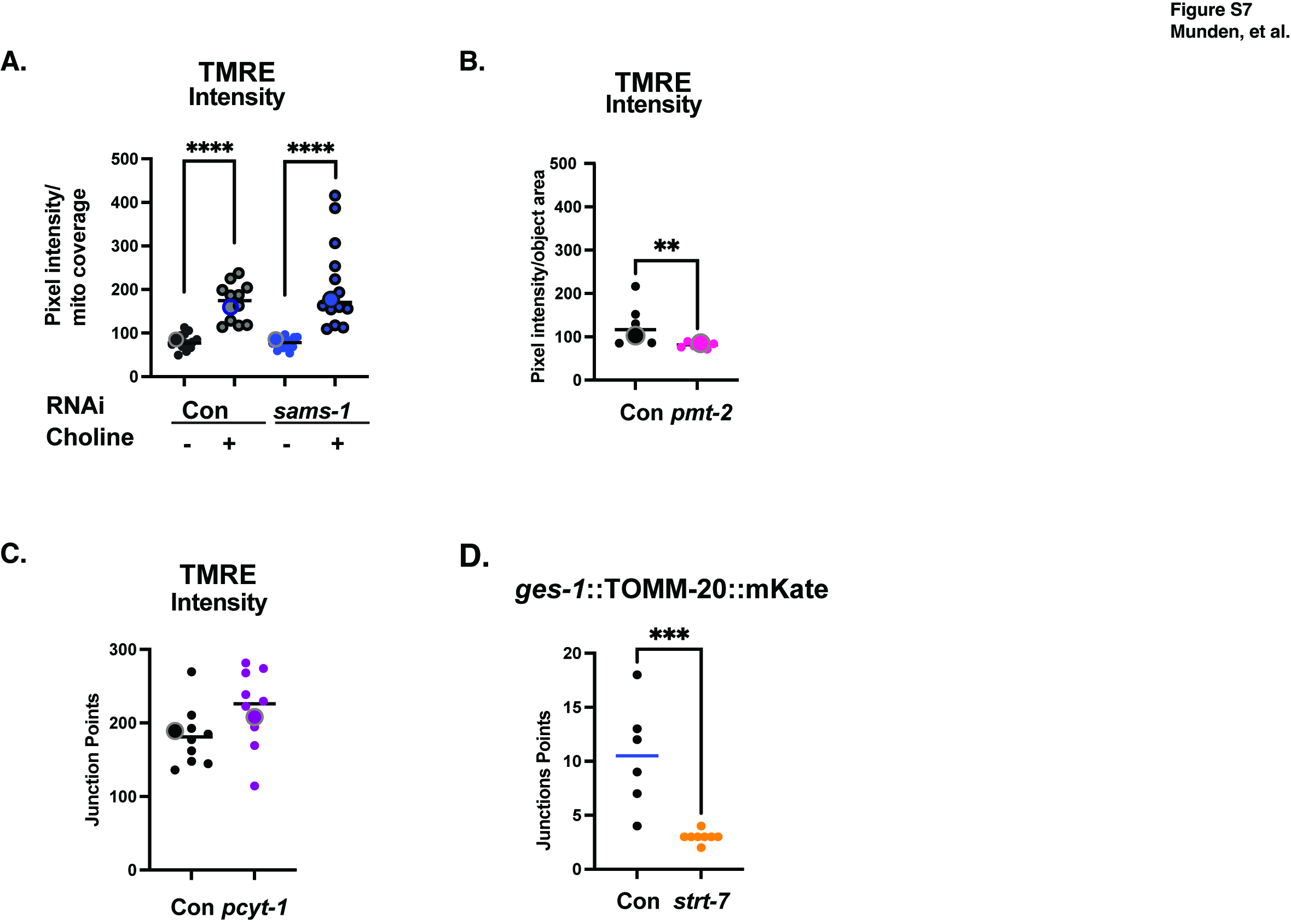

Supplement: S7 Fig — TMRE intensity comparison in wild-type and sams-1(RNAi) animals raised with and without dietary choline (A) or after pmt-2 (B), pcyt-1 (C) RNAi. (D) Quantitation of mitochondrial networks from intestinally expressed ges-1::TOMMM-20::mKate in control or strt-1(RNAi) treated animals. Significance calculated by the Mann–Whitney test is shown with * p < 0.01, ** p < 0.005, *** p < 0.001. Underlying data is in S1 Data. (TIF) [file pbio.3003075.s007.tif]

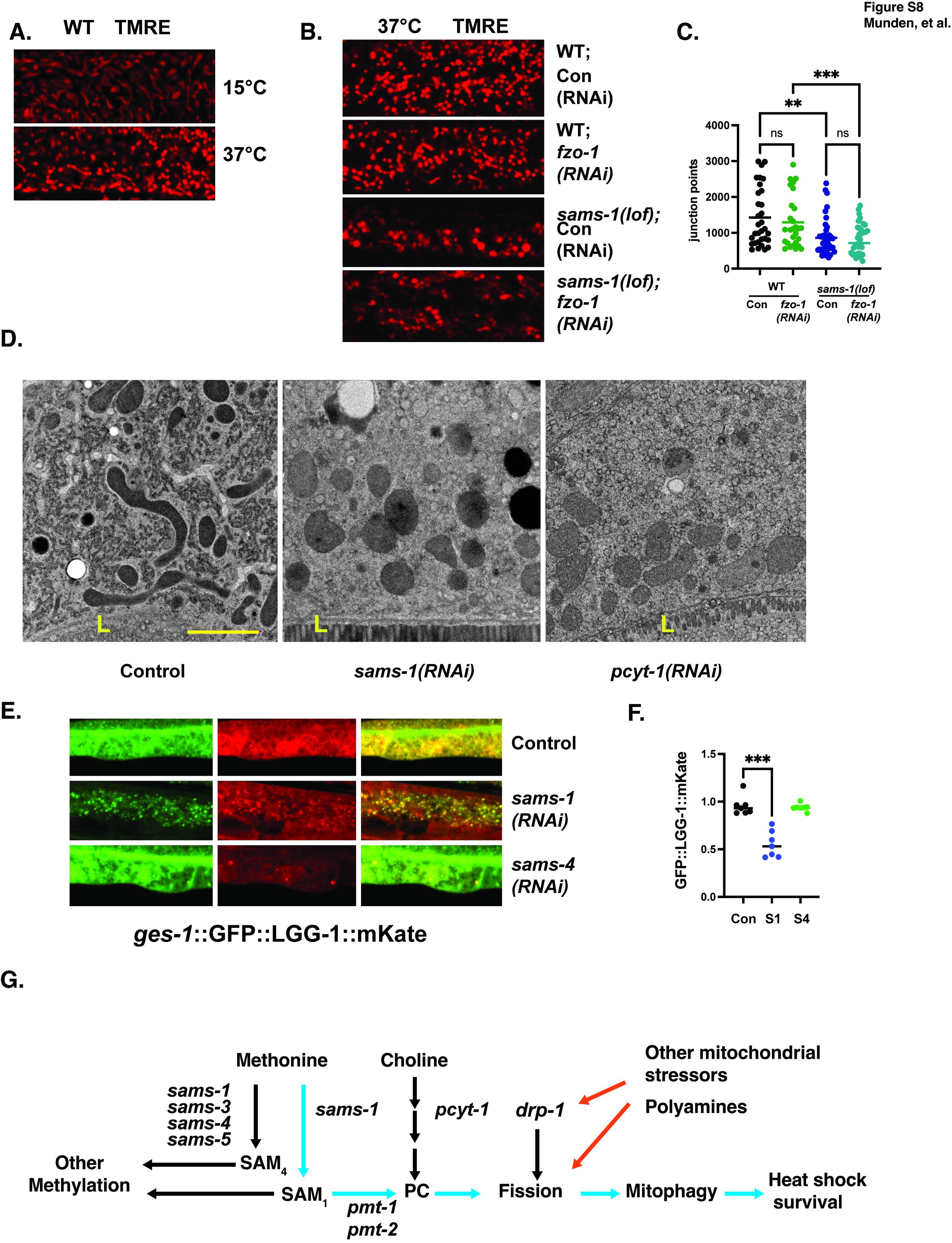

Supplement: S8 Fig — Spinning disc confocal projections of TMRE staining comparing basal and heat-shocked animals (A) or heat-shocked wild-type and sams-1(lof) animals exposed to fzo-1(RNAi) (B). Quantitation of junction points is in (C). (D) TEM images of Control, sams-1 or pcyt-1 RNAi animals comparing mitochondrial morphology, localization and localization within intestinal cells. Scale bar is 500 nm. (E) Confocal projections of animals with intestinal expression of an autophagy flux reporter, GFP::LGG-1::mKate [68] (E). Quantitation is in (F). (G) Model. Significance calculated by the Mann–Whitney test is shown with * p < 0.01, ** p < 0.005, *** p < 0.001. Underlying data is in S1 Data. (TIF) [file pbio.3003075.s008.tif]

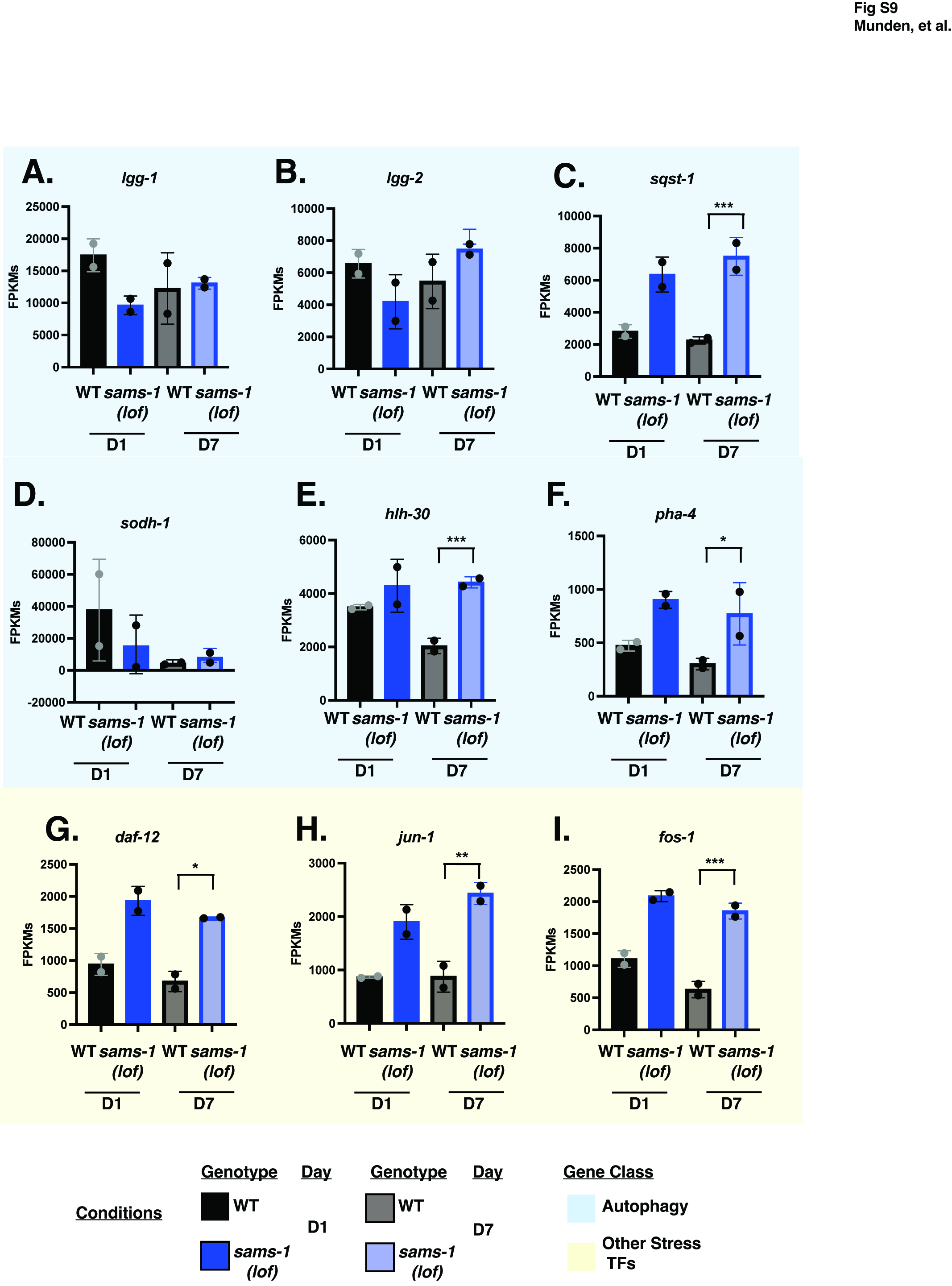

Supplement: S9 Fig — Column graphs of FPKMs from RNAseq for autogphagy–related genes (blue: A–F), other stress-related transcription factors (yellow: G–I). Whiskers encompass standard deviation and the p-adjust value calculated by Deseq2 shows significance including a false discovery rate with * p < 0.01, ** p < 0.005, *** p < 0.001. Underlying data is in S2 Table. (TIF) [file pbio.3003075.s009.tif]

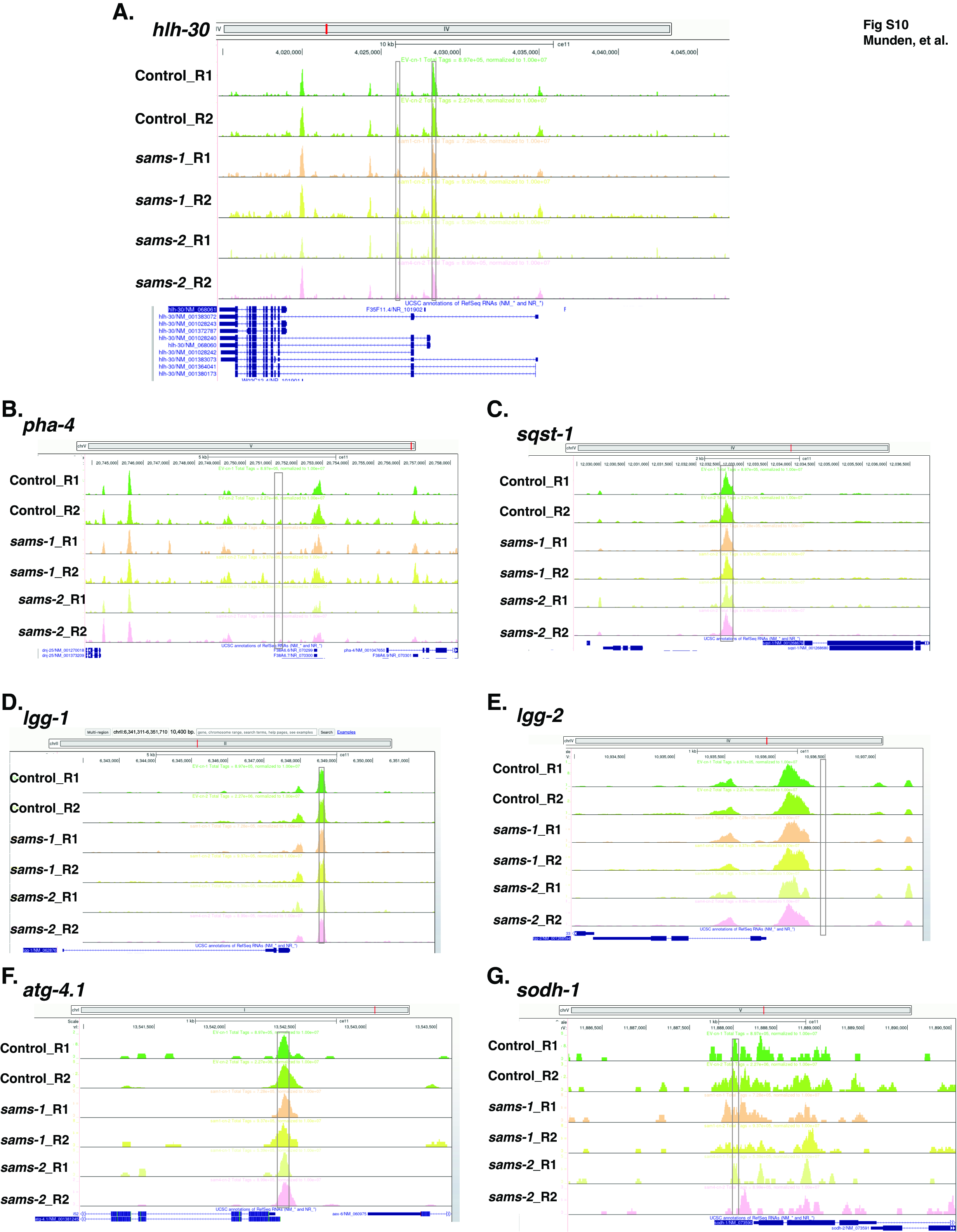

Supplement: S10 Fig — Locations for primer sets used in Lim and colleagues 2023 are boxed. (TIF) [file pbio.3003075.s010.tif]
