# Supplementary material for: Distinct S-adenosylmethionine synthases link phosphatidylcholine to mitochondrial function and stress survival
Source: PLoS Biol. 2025 Dec 1;23(12):e3003075. doi: 10.1371/journal.pbio.3003075 (PMC12680360; doi:10.1371/journal.pbio.3003075)
Supplement: S1 Raw Image — For top row, blot was cut below the 55kd marker. In the middle row the blot was cut below the 55kd Marker and was the same gel as the TBA-1 probed blot. In the bottom row, the blot was cut above the 55kd marker. Although these are from the same gel, the blot was cut in the middle. Therefore, a break was placed in the figure. This is the same gel as the ETFB-1 probed blot. Imaging was done on an iQuant 1500 system blot. (PDF) [file pbio.3003075.s017.pdf]

Figure 6C top row

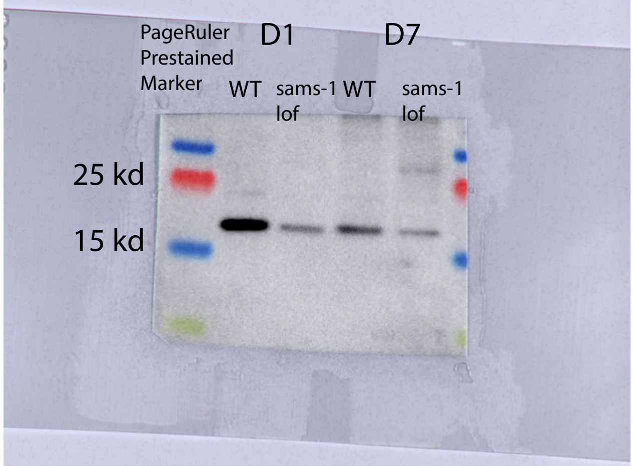

Blot was cut below the 55kd Marker.

Imaging was done on an iQuant 1500 system

Figure 6C middle row

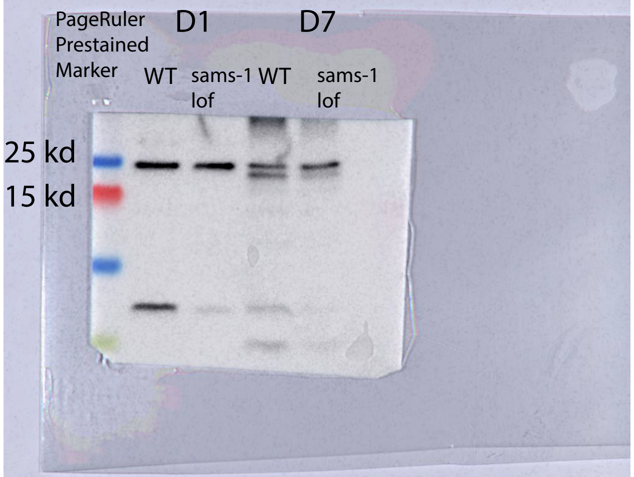

Blot was cut below the 55kd Marker.
This was the same gel as the TBA-1 probed blot.
Imaging was done on an iQuant 1500 system.

Figure 6C bottom row

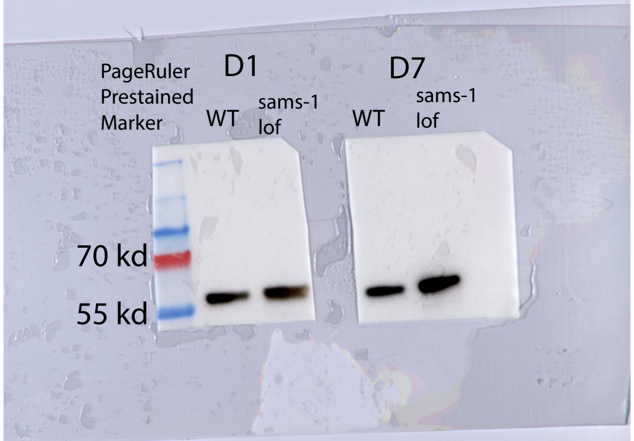

Blot was cut above the 55kd Marker.
Although these are from the same gel,
the blot was cut in the middle.
Therefore a break was placed in the figure.
This is the same gel as the ETFB-1
probed blot.
Imaging was done on an iQuant 1500 system
