# Supplementary material for: Distinct S-adenosylmethionine synthases link phosphatidylcholine to mitochondrial function and stress survival
Source: PLoS Biol. 2025 Dec 1;23(12):e3003075. doi: 10.1371/journal.pbio.3003075 (PMC12680360; doi:10.1371/journal.pbio.3003075)
Supplement: Attachment — Submitted filename: Response to reviewers_091825.pdf [file pbio.3003075.s020.pdf]

General response to reviewers:

We thank the reviewers for the careful reading of our study and for their comments. We also appreciate that their questions highlight some of the areas of great interest in the field of methyl donor metabolism and we have attempted to address their suggestions through experimentation and textual clarification. In particular, we appreciate the chance to add rigor to our RNA seq studies through completion of an additional set of aging RNA seq and qRTPCR. In order to better present these datasets, we have combined the original RNA seq data figures (Fig-4-6) into two figures (Fig4-5), moving the autophagy genes from Figure 5 to a new supplement (FigS9). The new data is presented in FigS4.

We also feel that the suggestion to address PC transport into the mitochondria has provided even more strength to our arguments regarding the SAM-PC-mitochondria link. Finally, we have added data showing *sams-1* mitochondria are functionally distinct from controls, using mitochondria IP'd from a *ges-1*::TOMM-20::mKate::HA strain in a Seahorse assay. This method was chosen because *sams-1* animals are much smaller than controls, and lack a germline; thus direct animal-animal comparisons are likely to measure mitochondrial number rather than function. To accommodate the new data in this section, we have split the original Fig7 into two figures (now Fig6 and Fig7), focusing Fig6 on SAM synthase specificity and Fig7 on the connection to PC. This also allows us to show additional quantitation of TMRE intensity and include the myo-3::TOMM-20::mKate that was previously in a supplemental figure.

We have supplied our responses below the reviewer comments in blue type.

Reviewer 1:

Major concerns:

-RNAseq data, especially C. elegans RNAseq data can be quite variable, as is very apparent in the PCA of Fig. 4B. It seems like the authors only conducted 2 replicates each of the RNAseq and with the exception of sams-1 RNAi at day 1, the other samples do not cluster very well. The clustering in Fig. 4C is definitely much better but I would highly recommend at least one more replicate is conducted to ensure the data is robust. This is especially important considering the many strong conclusive arguments the authors are making solely based on the RNAseq data without any experimental

validation of the data (e.g., that hsp-6 levels are unchanged in the sams-1 RNAi condition, directly in opposition to previously published hsp-6p::GFP data, arguing that this is an artifact of the transgene reporter; arguing against a role for sams-1 RNAi in induction of autophagy, etc.).

We agree that the robustness of the data could be improved with more replicates and addition of alternative approaches. We chose to address these questions by 1) repeating the RNA seq of the aging animals with three new replicates and analysis, 2) using qPCR to confirm RNA seq results on *sams-1* animals at D1 of adulthood, including a control which tested the requirement for *sams-1* during larval stages and 3) using qPCR to confirm RNA seq results at D1 and D7 of adulthood.

1) We have provided a new set of RNA seq data with three replicates (new figure S4). In this analysis, which has strong groupings of the Wild type and *sams-1(lof)* animals in the PCA plot (FigS4A), we observed similar effects on enrichment of mitochondrial gene expression in *sams-1* down regulated genes at D7 of adulthood (FigS4B), with similar effects shown on individual mitochondrial genes (FigS4 C-F). We also note that *hsp-4* levels are not altered (FigS4H), as in the previous analysis. The only difference in the levels of the autophagy genes between the two data sets was a 2 fold increase in *lgg-1* at D7 of adulthood. However, in D1 animals, where the comparison to Lim et al was made, the levels were unchanged. We apologize for not making this point clearer and have altered the text to point out that *hsp-4* , *hsp-6* or the autophagy genes did not change in any of our previous microarray, or 4 distinct sets of RNA seq studies (Ding, Cell Met, 2015, Ding et al. PLoS Genetics, Godbole, eLife 2023), therefore our conclusions were based on multiple independent studies.

We chose to use sufficient replicates to analyze the new RNA seq data on its own, rather than complete an additional replicate to analyze with the previous results because of the precision needed to insure identical biological conditions in the aged population.

2) While RNA seq provides the advantage of normalization across the entire genome, the data in Chen et al. was qRT-PCR, therefore it was important to compare similar assays for the mitoUPR and autophagy genes. As in our RNA

seq studies, the endogenous genes for the mitoUPR or autophagy regulators were not significantly changed.

The reviewer also brings up an important point that we have worked to clarify both experimentally and in the text regarding *sams-1* and autophagy. We agree that increased autophagy is occurring in *sams-1* animals, but find no support for a mechanism based on H3K4me mediated effects on autophagy gene regulation. In order to address this via our qPCR assays, we took advantage of this format to compare *sams-1* animals in standard media conditions with those that had been provided with choline to rescue PC production during larval stages. This allows us to “rescue” PC-dependent effects *sams-1* during larval stages and induce phenotypes in adulthood. However, choline does not rescue H3K4me3 levels in intestinal nuclei (Ding, Cell Met 2015), which allows us to separate histone methylation from PC effects in animals at all stages. Notably, choline rescued sams-1 animals did not show changes in mitoUPR or autophagy genes. H3K4 methylation based mechanisms would be expected to be revealed in these assays.

In order to clarify the data and discussion regarding the absence transcriptional regulation of autophagy, we have moved this to the last section of the paper, where it is presented as an alternative hypothesis to PC-based effects.

-To make an argument that lifespan extension of sams-1lof is independent of autophagy based on RNAseq data is incorrect. While many autophagy-related genes (ATGs) are transcriptionally upregulated during prolonged autophagy, short-term or acute activation of autophagy can occur primarily through post-translational modifications and signaling cascades. Therefore, it is entirely possible that even with a lack of autophagy gene activation, there is significant autophagy activation and that the lifespan extension is dependent on autophagy. Either this conclusion needs to be removed or experimentally tested (e.g., disrupt autophagy and show that lifespan extension still occurs in sams-1lof).

We agree with the reviewer and have revised our text to clarify this issue; we regret that our original text was not clear.

Several aspects of the *sams-1* phenotype have been ascribed to roles in gene regulation through epigenetic changes from autophagy (Lim, et al) to changes in

mitochondrial morphology (Wei and Ruvkun). However, our studies clearly link the mitochondrial changes in *sams-1* animals to drops in PC. Lim et al show increases in LGG-1 puncta and a partial reduction in *sams-1(lof)* animals after *bec-1*/Beclin RNAi, suggesting increases in autophagy and contribution to lifespan. Using different strains, we also show increases in LGG-1 puncta and degradation of GFP::LGG-1, supporting the notion that autophagy is increased in *sams-1* animals.

We agree with the reviewer that these events are unlikely to be due to direct transcriptional/histone modification-mediated mechanisms. We have moved discussion of autophagy gene regulation and also worked to distinguish our work in this study, which focuses on the biological context of heat shock from other studies that are using lifespan as a phenotypic output.

-Because there is no strain list available, I am not sure which TOMM-20::mKate strain was being used. Was this an endogenously tagged TOMM-20 or an overexpression? If it is not endogenously tagged, the authors cannot use this as a proxy to measure mitochondrial mass since there is no way to distinguish between changes in transgene expression or mislocalization of an overexpressed mitochondrial protein. The authors either need to remove this or experimentally validate in an appropriate way (e.g., biochemically by probing for an endogenous mitochondrial-localized protein like porin, or quantifying mtDNA).

-If the authors are arguing a marked decrease in mitochondrial mass based on their TOMM-20::mKate, why is TMRE staining in Fig. 7C not lower? However, TMRE staining in Fig. 7F for sams-1 is actually lower, then it's higher in Fig. 7H? This is extremely confusing why the sams-1 RNAi vs. control is so dramatically different in each figure. Why does sams-4 RNAi have lower signal? Are all these differences because of decreased membrane potential or mitochondrial mass or both? These inconsistencies need to be addressed.

We thank the reviewer for pointing out the omission of the strain list, which we now provide (Data S5) and for the thoughtful comments on improving our interpretation of the mitochondrial imaging.

1) TOMM-20: Regarding the TOMM-20 expression levels, we used SJZ204, where *tomm-20* is expressed from a *ges-1* promoter in a MosCI locus, thus there is only one additional copy of *tomm-20*. Estimates from modEncode show that *ges-1* is expressed around 13 FPKMs in adult animals, whereas *tomm-20* is about 120. While the fact that *ges-1* is intestine specific and *tomm-2*0 would have a more global profile could account for some of these differences, the *ges-1* promoter would not appear to drive expression at a significantly higher level than the endogenous gene.

To more directly answer the question regarding levels of mitochondrial proteins, we have used immunoblots to examine levels of endogenous TOMM-20 along with another mitochondrial protein ETFB-1, examined mitochondrial DNA levels and the mitochondrial footprint of the TMRE (new Fig7C, D). The immunoblots confirmed that endogenous TOMM-20 levels decrease at D1 and D7 animals after sams-1 RNAi, along with showing drops in TOMM-20 during aging, paralleling our RNA seq data and the confocal imaging showing decreases in TOMM-20. However, ETFB-1, a flavoprotein connected to complex 2, mitochondrial DNA and TMRE mitochondrial footprint were similar between sams-1 and control animals at D1 of adulthood.

This suggests that increased interactions between mitochondria and autophagosomes leading to degradation are balanced at D1 of adulthood, consistent with an impact on increased survival after heat stress, and that loss of mitochondrial gene expression and lowered levels of some proteins is a later step.

2) TMRE: The reviewer also points out that the images we selected for the TMRE panels could be confusing to the reader and our discussion of the TMRE results should include more information. We have taken two steps to address these issues. First, we reselected images for each panel to provide a better example of a representative animal. In each case, we marked the point in the quantitation corresponding to the image with a larger, contrasting point. Thus, the representative nature of each image is more apparent. We also note that the data in Fig7 comparing the SAM synthases and the choline rescue of sams-1 animals, now show in Fig8, were all performed in the same experiment. We selected distinct images for these panels, which we hoped to clarify by highlighting location of the image in the quantitation.

Next, we included more information from our TMRE quantitation to clarify the reviewer's additional points. The mitochondrial footprint shows significant decrease across the *sams-1* animals, which are not changed after RNAi of the other SAM synthases (new Fig7G). Notably, the TMRE intensity does not change, suggesting that membrane potential has not altered in the D1 animals at the time of our heat shock assays. This is a critical point, as it allows us to compare morphological changes without the confounding issues occurring if levels also change. The reviewer's questions also alerted us to the need to clarify distinctions between changes in mitochondrial morphology and overlap with autophagosomes at D1 of adulthood, which we suggest impact heat shock survival and from impacts on mitochondria in aging animals, thus we have worked to improve the text to better differentiate these biological contexts.

-The authors are arguing that sams-1lof animals do not have higher thermotolerance in fzo-1 depletion because they say sams-1lof already has fragmented mitochondria. If this is the case, then is drp-1 RNAi not reducing thermotolerance because drp-1 knockdown cannot result in increased fusion of mitochondria in the sams-1lof animal? Please perform the imaging of fzo-1/sams-1lof and drp-1/sams-1lof to ensure that the conclusions about linking mitochondrial fragmentation to thermotolerance are actually valid.

Reviewer 1 brings up the point that we based our conclusions on the interactions of *sams-1* and mitochondrial morphology were based on genetic interactions and should be supplemented with imaging. To address this point, we used TMRE to confirm previously reported changes in mitochondria after heat shock (see Chen JCB 2021) and observed the reported fragmentation. Next, we asked if loss of *fzo-1* could increase additional fission and found that levels in control or *sams-1(lof)* animals did not decrease. We have also altered the text to more clearly state our hypothesis that low PC causes alterations in mitochondria in *sams-1* animals. Low PC has been shown in the case of lipid droplets to cause formation of larger, more rounded structures, as the membranes lack the capacity to make complex bends. Thus, the phospholipid balance would constrain the morphology and render it less sensitive to other mechanisms of morphological control

-Please include a strain list. This is essential to ensure that the experiments can be reproduced by future studies.

The strain list is now supplied (DataS5).

Minor concerns:

-Page 17 - where Fig. 7C, and D are referenced, RNAi should have a capital A.

-For referencing hsp-6p::GFP and hsp-4p::GFP please put the "p" to indicate promoter to prevent confusion with the HSP-6::GFP and HSP-4::GFP translational reporters.

These have been corrected.

Reviewer 2:

(1) Figs. 1E, 2B: Heat shock stress induced high accumulation of SAM in wild-type, sams-1, and sams-4 mutants. The reason for this is that heat shock stress activates SAM synthesis, but it is unclear whether SAM synthesis is actually promoted or not. The link between increased methylation and increased SAM synthesis is not clear, especially since the same group's 2023 paper published in eLife showed that sams-4 mutants exhibited no increase in H3K4Me3 levels upon heat shock stress. The authors should examine the flux analysis of SAM when heat shock stress is applied in wild strains, sams-1, and sams-4 mutants, and what happens to the accumulation of SAM when heat shock stress is administered in the sams-1 mutant under knockdown of sams-4.

Reviewer 2 raises multiple interesting questions about SAM synthesis and points out places where we should have been clearer in our text. The most central issue of their point is that understanding SAM flux is critical. That undertaking would constitute a separate study, therefore, we have added to our discussion acknowledge this importance and to better present alternative interpretations of our data.

In our study, Godbole et al. eLife 2023, we show that SAM increases during heat shock and that increased H3K4me3 in *sams-1(lof)* animals requires a distinct SAM synthase, *sams-4*. However, that genetic requirement does not imply SAM

synthesis is regulated, only that synthase is necessary. As *sams-4* and the H3K4 methyltransferase *set-16* (Ding, et al) is also necessary for survival during heat shock, our interpretation from our previous work is that SAMS-4 supplies a subset of H3K4me3 that is needed for animals to survive this stress. In the present study, we have compared *sams-1* and sams-4 metabolomes, but then focused on changes in *sams-1* animals that could explain its ability to survive better after heat shock. We agree the flux experiment would be exciting, but development of this approach in *C. elegans* would be beyond our scope for this study. We have added text to acknowledge this point.

(2) Figs. 1, 2: Did spermidine levels change in metabolome analysis? It has been shown that spermidine contributed to lifespan extension in Caenorhabditis elegans (Nat Cell Biol. 2009 doi: 10.1038/ncb1975. Epub 2009 Oct 4. Tobias Eisenberg et al.). Therefore, does impairing spermidine synthesis affect the phenotype of sams-1 mutant strains? It has been reported that a decreased methionine level promoted choline synthesis and decreased SAM synthesis, which in turn enhanced putrescine and spermidine synthesis (Biomolecules. 2023 doi: 10.3390/biom13030471. Sayaka Harada et al.). Could the same thing be happening in the sams-1 mutant strain?

(3) Figs. S2B, S2C: Why do sams-1 mutants accumulated high levels of polyamines despite decreased accumulation of SAM?

As reviewer 2 points out, SAM is important for polyamine production. In our targeted metabolomics, we did detect spermidine, but the only statistically significant change we found was between *sams-4* basal and heat shocked animals. We have added the spermidine individual plot to FigS2 along with providing a box highlighting metabolites linked to polyamine and arginine synthesis. While there are changes in several metabolites in *sams-1* animals or after heat shock, there were no cases where these metabolite changes correlated with both lack of *sams-1* and survival in heat shock, reducing confidence that they had direct links to the mechanism.

Reviewer 2 brings up an interesting idea with the Harada publication, which shows a link from choline to SAM and PC production. However, mammalian and *C. elegans* metabolism appear to have an important difference in a key part of this pathway; *C. elegans* does not contain the betaine hydroxy methyltransferase necessary for the pathway to convert choline all the way to SAM. Kang et al (Nat Cell Bio, 2024) suggest *metr-1* could perform a similar reaction, however, we found that choline is insufficient to rescue H3K4me3 immunostaining in *sams*-1

intestinal cells (Ding, Cell Met 2015), demonstrating that if *metr-1* could convert betaine-derived from choline to SAM, that pool is not able to generally rescue H3K4 methylation. As we also found that adding choline to sams-1(RNAi) animals robustly rescues PC (Ding, Cell Met 2015), the most likely mechanisms for choline action on *sams-1* animals by supporting PC production in the Kennedy pathway.

Regarding the question of why there are not more changes in polyamines in *sams-1* animals, we hypothesize that components of the media may provide rescue for some of these metabolites. Macrae et al (J Biochem, 1998) find that media components are capable of rescuing the ocd-1 mutation during development, explaining the limited phenotypes of this mutation on normal growth media.

We agree with the reviewer that the link between polyamines, SAM and mitophagy is intriguing (see Giovannetti, iScience 2024). In our studies, we have focused on the clear links with phosphatidylcholine levels. However, mitochondrial morphological responses are a general response to stress and may be stimulated by multiple mechanisms. To better acknowledge this complexity, we have added polyamines to our diagram in FigS8G as an independent inducer of autophagy.

(4) What is the mechanism by which abnormal PC synthesis affects MT function? Have the authors investigated the possibility that MT function is affected via lipid exchange by the ER-mitochondria connection?

Phosphatidylcholine is a major component of the lipid bilayer. Its level can have a major influence on membrane dynamics and the ability for a membrane to curve; however, it could also act through effects on signaling. PC can be cleaved by phospholipase D to generate phosphatidic acid or choline, each of which could potentially affect downstream pathways. As reviewer 2 suggests, examining PC transport into the mitochondria would be an advantageous way to determine if the effects of low PC are acting directly on mitochondrial membranes. We focused on two genes, *vps-13D*, which can move PC between organelles and *strt*-7/STARD7, a flippase that can move PC between the inner and outer mitochondrial leaflets and has previously been linked to changes in

mitochondrial morphology (Decker, Cell Metab 2024). The Alkema lab has recently shown that reductions in vps-13D alter mitochondrial morphology in body wall muscle cells (Yin, et al G3 2025). We observed similar changes with TMRE staining of hypodermal cells (new Fig8 I). STARD7 mutations in human cells are associated with changes in PC within the mitochondria (Saita, EMBO J 2018). In *C. elegans*, we identified two STAR domain family proteins, one of which, *strt-7*, contains a mitochondrial targeting sequence and is expressed predominantly in the intestine. Using the intestinally expressed TOMM-20::mKate, we observed moderate changes in morphology as mitochondrial networks became less connected. Thus, these experiments provide important additional evidence supporting the link between low PC and the changes in mitochondrial morphology in *sams-1* animals.

(5) What is the mechanism by which SAMs synthesized by SAMS-1 and SAMS-4 are primarily used for metabolism and methylation, respectively? Is it possible that the expression of SAMS-4 in the gonads affects methylation? Experiments with L1, which is unaffected by the gonads, may help clarify this.

In this study, we have focused on building a link between the survival of *sams-1* animals after heat shock to metabolic changes, such a lowering in some mitochondrial metabolites, aging -dependent changes in mitochondrial gene expression and alterations in mitochondrial morphology that might enhance survival through mitophagy. These effects are specific to *sams-1*, providing a critical mechanism for the SAM synthase specific effects we have observed. We agree that the question of how SAM is provisioned to metabolism or protein methylation is a critical one in the field. However, our first step in answering these questions was to define the mechanisms linking *sams-1* to the mitochondrial changes, where we show that PC production is sufficient to drive these phenotypes and that they do not occur after *sams-4* RNAi. Finally, *sams-1(lof)* or RNA animals have small germlines due to the need for PC to secrete the yolk proteins (Fanelli, Sci Adv 2023) and form the membrane of the syncytial germline. Thus, germline-intrinsic SAMS-4 would be likely to have minimal effects in these animals.

The question about examination of early larval and germline effects from SAMS-4 brought up by reviewer 2 is thoughtful and highlights the complexity of

understanding how and where SAM is produced how that might be linked to phenotypes. While we feel that additional experimentation with animals of a different developmental stage may reach beyond the present scope, at the discretion of the editor and reviewers, we can discuss potential effects of germline SAM synthase expression in more detail.
